# A Single-Domain Response Regulator Functions as an Integrating Hub To Coordinate General Stress Response and Development in Alphaproteobacteria

**DOI:** 10.1128/mBio.00809-18

**Published:** 2018-05-22

**Authors:** C. Lori, A. Kaczmarczyk, I. de Jong, U. Jenal

**Affiliations:** a Focal Area of Infection Biology, Biozentrum, University of Basel, Basel, Switzerland; National Cancer Institute

**Keywords:** *Caulobacter*, alphaproteobacteria, bow-tie, general stress response, phosphorylation, regulation of gene expression, two-component system

## Abstract

The alphaproteobacterial general stress response is governed by a conserved partner-switching mechanism that is triggered by phosphorylation of the response regulator PhyR. In the model organism Caulobacter crescentus, PhyR was proposed to be phosphorylated by the histidine kinase PhyK, but biochemical evidence in support of such a role of PhyK is missing. Here, we identify a single-domain response regulator, MrrA, that is essential for general stress response activation in C. crescentus. We demonstrate that PhyK does not function as a kinase but accepts phosphoryl groups from MrrA and passes them on to PhyR, adopting the role of a histidine phosphotransferase. MrrA is phosphorylated by at least six histidine kinases that likely serve as stress sensors. MrrA also transfers phosphate to LovK, a histidine kinase involved in C. crescentus holdfast production and attachment, which also negatively regulates the general stress response. We show that LovK together with the response regulator LovR acts as a phosphate sink to redirect phosphate flux away from the PhyKR branch. In agreement with the biochemical data, an *mrrA* mutant is unable to activate the general stress response and shows a hyperattachment phenotype, which is linked to decreased expression of the major holdfast inhibitory protein HfiA. We propose that MrrA serves as a central phosphorylation hub that coordinates the general stress response with C. crescentus development and other adaptive behaviors. The characteristic bow-tie architecture of this phosphorylation network with MrrA as the central knot may expedite the evolvability and species-specific niche adaptation of this group of bacteria.

## IMPORTANCE

Two-component systems (TCSs) consisting of a histidine kinase and a cognate response regulator are predominant signal transduction systems in bacteria. To avoid cross talk, TCSs are generally thought to be highly insulated from each other. However, this notion is based largely on studies of the HisKA subfamily of histidine kinases, while little information is available for the HWE and HisKA2 subfamilies. The latter have been implicated in the alphaproteobacterial general stress response. Here, we show that in the model organism Caulobacter crescentus an atypical FATGUY-type single-domain response regulator, MrrA, is highly promiscuous in accepting and transferring phosphoryl groups from and to multiple up- and downstream kinases, challenging the current view of strictly insulated TCSs. Instead, we propose that FATGUY response regulators have evolved in alphaproteobacteria as central phosphorylation hubs to broadly sample information and distribute phosphoryl groups between the general stress response pathway and other TCSs, thereby coordinating multiple cellular behaviors.

## INTRODUCTION

All living organisms must constantly monitor their environment to ensure survival and successful reproduction. In bacteria, adaptive responses often involve motility or chemotaxis, the formation of surface-grown multicellular biofilms, or general and specific stress responses. Through altered behavior or physiological states, bacterial cells can withstand or escape potentially harmful or suboptimal conditions that endanger their fitness. However, adaptive responses interfere with normal development or proliferation, and conversely, specific developmental stages may shape an organism’s ability to tolerate and respond to environmental changes. For instance, the ability of Caulobacter crescentus to escape or withstand unfavorable conditions changes during the reproductive cycle. C. crescentus has a dimorphic lifestyle that, upon division, produces a motile and a sessile daughter (1). The motile swarmer (SW) cell is equipped with a flagellum and is able to perform chemotaxis but remains in a replication-incompetent state. To proliferate, the SW cell needs to differentiate into a sessile stalked cell, a process during which it loses its flagellum, synthesizes an adhesin called the holdfast, and initiates replication and cell division. Intriguingly, the ability of C. crescentus to survive stressful conditions depends on the cell cycle stage (2). Moreover, cells experiencing stress or suboptimal growth conditions respond by adjusting their development and cell cycle progression (3). For instance, when starved for carbon, cells respond by blocking cell cycle progression and chromosome replication (4, 5). In contrast, C. crescentus cells experiencing heat or ethanol stress respond by overreplicating their chromosomes (5). While these responses are thought to increase bacterial survival, the underlying molecular mechanisms coordinating the stress response with developmental or reproductive processes remain largely unknown.

Bacterial signal transduction is dominated by two-component phosphorylation cascades (6). Generally, a histidine kinase undergoes autophosphorylation on a conserved histidine residue upon perception of a specific external or internal stimulus. The phosphoryl group is then transferred to a conserved aspartate residue of the receiver (Rec) domain of a cognate response regulator. Rec modification in turn controls the activity of various response regulator output domains (7). A subclass of response regulators, called single-domain response regulators (SDRRs), lacks a dedicated output domain, comprising only the phosphoryl-accepting Rec domain (8). These proteins are thought to act by directly interacting with other proteins and allosterically modulating their activity (9, 10) or as shuttles or sinks, transferring phosphoryl groups between phosphorelay components or draining phosphate away from histidine kinases (11–14). The C. crescentus genome encodes a total of 20 SDRRs, a large fraction of which interact with the flagellar motor similar to the canonical CheY protein in Escherichia coli (15). Two SDRRs, DivK and CpdR, are members of a complex regulatory network controlling the activity of the cell cycle regulator CtrA, a central response regulator mediating C. crescentus proliferation and behavior (16–18). While DivK acts as an allosteric regulator of several cell cycle kinases that are positioned upstream of CtrA (10, 19–21), CpdR serves as a protease adapter to control cell cycle-dependent degradation of CtrA (18, 22).

**FIG 1:**
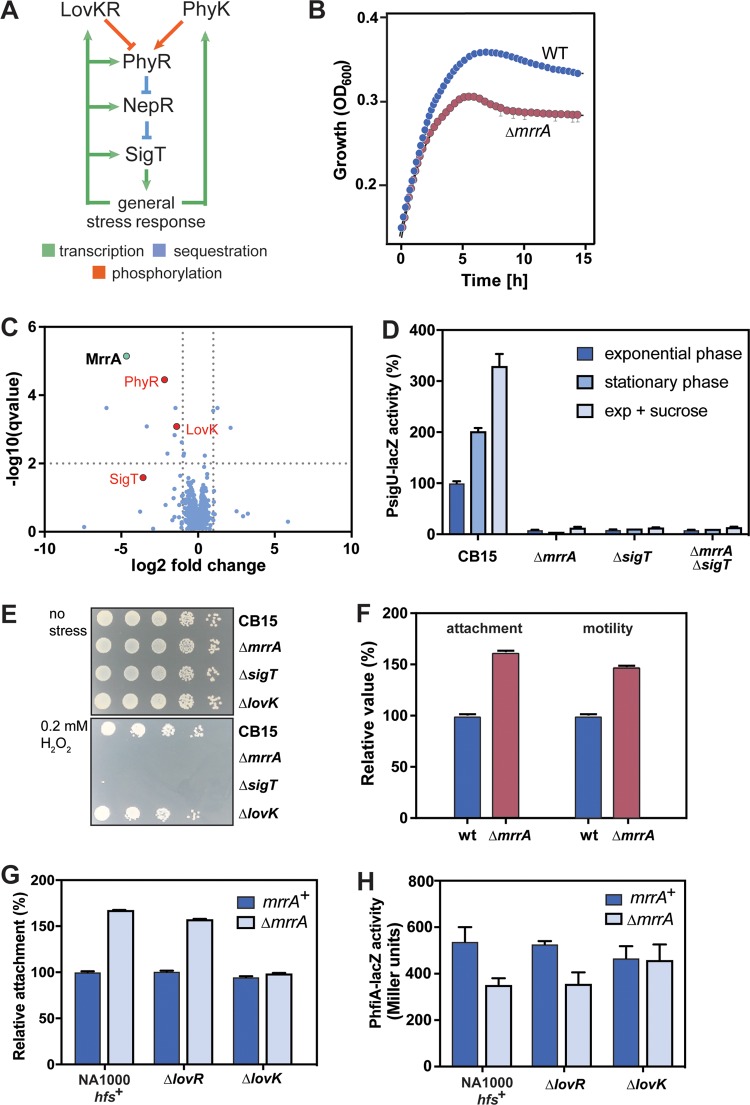
MrrA controls C. crescentus behavior and stress response. (A) Schematic representation of the general stress response pathway in alphaproteobacteria. (B) An Δ*mrrA* mutant shows decreased growth upon entry into stationary phase. Growth was measured in 96-well plates by periodically monitoring optical density at 660 nm. WT, wild type. (C) Comparison of proteomes of C. crescentus wild type and *ΔmrrA* mutant. On the *x* axis, log_2_ fold changes of the *ΔmrrA*/wild-type (WT) ratios are plotted, and on the *y* axis, log_10_(*q* value) data are plotted. Red dots highlight targets that were significantly downregulated and were suggested to be involved in the general stress response. MrrA is highlighted in green. (D) MrrA is required for SigT-dependent gene expression. LacZ reporter fusions were used to determine the activity of the SigT-dependent *sigU* promoter. Cells were grown in PYE or PYE supplemented with 150 mM sucrose with two biological and several technical replicates being used per strain and condition. Error bars indicate the standard deviation. (E) MrrA is required for efficient survival under stress. Cells were grown to exponential phase in minimal medium with xylose (M2X) and stressed using 0.2 mM H_2_O_2_ for 1 h. Serial 1:10 dilutions are shown. (F) MrrA controls C. crescentus surface attachment and motility. Relative values of overall attachment and motility are shown and were determined as outlined in Materials and Methods. wt, wild type. (G) LovK is essential for the surface attachment repression of MrrA. Relative values of overall attachment are shown. (H) MrrA increases *hfiA* expression in a LovK-dependent manner. Cells harboring *hfiA*-*lacZ* reporter fusions were grown in PYE, and β-galactosidase activities were determined as described in Materials and Methods. For all experiments shown in this figure, two biological replicates and several technical replicates were used.

Functional information is available for one additional member of the SDRR family in C. crescentus, LovR. LovR is encoded in the *lovKR* operon, with LovK considered to be the cognate kinase of LovR. The LovK/LovR two-component system was proposed to control C. crescentus surface attachment in response to blue light by promoting the production of adhesive holdfast (23, 24). More recently, the LovKR proteins were shown to also negatively regulate the general stress response, an adaptive response to a diverse range of adverse environments and important for survival under harmful conditions (25). The alphaproteobacterial general stress response is conserved in essentially all free-living members of this class and is controlled by a partner-switching mechanism involving the sigma factor SigT (or σ^EcfG^), the anti-sigma factor NepR, and the response regulator and anti-anti-sigma factor PhyR (26–28) (Fig. 1A). In the absence of stress, PhyR is dephosphorylated and NepR interacts with SigT, preventing the sigma factor from productive interaction with RNA polymerase. When cells experience stress, PhyR is phosphorylated and binds NepR, resulting in SigT release and the activation of its target genes. While in C. crescentus PhyK was proposed to be the major histidine kinase of PhyR, LovK and LovR were proposed to play a role in PhyR dephosphorylation (25, 29). The mechanistic details of this process have remained elusive.

Based on the findings that SDRRs play central roles in C. crescentus development and physiological adaptation, we set out to genetically characterize select SDRRs in this organism. Here, we present evidence that the SDRR MrrA is an important regulator of developmental processes such as motility and attachment and that it controls cell behavior through LovK. Importantly, MrrA is also a central component of the C. crescentus general stress response that directly impacts PhyK and PhyR phosphorylation. Our results demonstrate that MrrA shuttles phosphoryl groups from a range of upstream kinases to both LovK and PhyK, which serve as histidine phosphotransferases. Based on these findings, we postulate that MrrA serves as a central phosphorylation hub that coordinates developmental processes with the general stress response in this organism.

## RESULTS

### MrrA is a response regulator that controls development and the general stress response.

To functionally characterize SDRRs in C. crescentus, in-frame deletions were generated in the respective genes (CC0630, CC2576, CC3015, and CC3286). These four SDRRs were chosen from a total of 20 SDRRs in C. crescentus based on the prediction that they are not involved in chemotaxis and had not been functionally characterized before (10, 15, 18, 23, 25). Whereas mutations of CC0630, CC2576, or CC3286 showed no apparent phenotype in the assays tested, a strain lacking CC3015 showed several behavioral and growth defects, based on which we renamed this protein MrrA for multifunctional response regulator A. When grown in a complex medium (peptone-yeast extract [PYE]), the Δ*mrrA* strain showed wild-type-like growth in exponential phase but entered stationary phase prematurely (Fig. 1B). This suggested that the Δ*mrrA* mutant may lack the ability to cope with certain forms of stress associated with stationary phase. To better understand the mechanisms provoking this phenotype, we compared the proteomes of C. crescentus wild-type and Δ*mrrA* strains. Strong reductions in protein abundance in the *mrrA* mutant were observed for central components of the general stress response pathway, including PhyR, NepR, and SigT, as well as for proteins that were previously identified as targets of SigT, including the histidine kinase LovK (Fig. 1C; see also Table S1 in the supplemental material) (4, 25). Because the core components of the general stress response are subject to autoregulation (Fig. 1A) (26, 28), these changes suggested that the Δ*mrrA* mutant failed to induce the general stress response under these growth conditions. This idea is consistent with the observation that the relative losses of fitness of an *mrrA* and a *sigT* mutant correlate for a range of different conditions (Fig. S1A) (30). To verify a role of MrrA in the general stress response, we made use of a *sigU*-*lacZ* reporter fusion, the activity of which strictly depends on the sigma factor SigT (25). In C. crescentus wild type, SigT was active in exponential phase and induced under osmotic stress or upon entry into stationary phase. In contrast, the Δ*mrrA* mutant showed no SigT activity irrespective of growth phase and stress applied (Fig. 1D), arguing that MrrA is indispensable for the activity of SigT. In line with this notion, the Δ*mrrA* strain showed a 1,000-fold reduction in survival compared to wild type when challenged by oxidative stress, essentially phenocopying a *sigT* null mutant (Fig. 1E).

10.1128/mBio.00809-18.1 FIG S1  (A) Comparison of the relative fitness of an Δ*mrrA* and a Δ*sigT* mutant under different conditions, each represented by an individual dot (30) (http://fit.genomics.lbl.gov/cgi-bin/compareGenes.cgi?orgId=Caulo&locus1=CCNA_03110&locus2=CCNA_03589). (B) Numbers and size of holdfast-mediated rosette structures are increased in an Δ*mrrA* mutant. Rosettes were analyzed in randomly chosen samples of exponentially growing cultures of wild type and the Δ*mrrA* mutant. (C) Comparison of c-di-GMP concentrations in C. crescentus wild type and an Δ*mrrA* mutant strain as determined by mass spectrometry analysis (see Materials and Methods). Download FIG S1, PDF file, 0.6 MB. Copyright © 2018 Lori et al. 2018 Lori et al. This content is distributed under the terms of the Creative Commons Attribution 4.0 International license.

10.1128/mBio.00809-18.4 TABLE S1  Identification of potential MrrA interaction partners. (A) Selected hits obtained by coimmunoprecipitation analysis of a strain expressing an *mrrA*-3×FLAG allele using anti-FLAG antibodies to pull down potential interaction partners. Total spectral counts obtained are compared to counts obtained with a control strain with untagged MrrA. (B) Hits obtained with a yeast two-hybrid (Y2H) analysis screen that failed to not show autoactivation and robustly grew after restreaking on selective plates. (C) Protein hits obtained from the whole-proteome comparison between wild type and an Δ*mrrA* strain. Only proteins that were minimally 4-fold down- or upregulated are shown. Download TABLE S1, PDF file, 0.1 MB. Copyright © 2018 Lori et al. 2018 Lori et al. This content is distributed under the terms of the Creative Commons Attribution 4.0 International license.

The Δ*mrrA* mutant also displayed increased surface attachment and increased spreading on semisolid agar plates (Fig. 1F), the latter of which requires an intact flagellar machinery and chemotactic behavior. This indicated that MrrA, directly or indirectly, inhibits both motility/chemotaxis and holdfast-dependent attachment, two behaviors that are usually regulated inversely (31). A role for MrrA in holdfast production was further supported by the observation that the Δ*mrrA* mutant showed a strong increase in the number and size of rosettes, characteristic holdfast-mediated C. crescentus cell aggregates (Fig. S1B) (23, 25). Because both motility and attachment are regulated by the second messenger c-di-GMP (31), we determined c-di-GMP levels in the Δ*mrrA* strain but found no significant differences compared to wild type (Fig. S1C). Holdfast biogenesis is also regulated by the holdfast inhibitor protein HfiA, which directly binds to and inhibits HfsJ, an essential component of the adhesive polysaccharide export machinery (32). Moreover, LovK and LovR were shown to regulate C. crescentus surface attachment via the expression of HfiA (32). Epistasis experiments revealed that the effect of MrrA on attachment depended on LovK but not on LovR (Fig. 1G). Likewise, *hfiA* expression was reduced in the Δ*mrrA* mutant, an effect that was dependent on LovK but not on LovR (Fig. 1H). These results support the notion that MrrA acts upstream of LovK to control attachment via the modulation of *hfiA* expression.

In sum, these results revealed MrrA as a pleiotropic regulator affecting motility, attachment, growth, and survival under stress conditions. In particular, our data indicated that MrrA is an essential component of the alphaproteobacterial general stress response in C. crescentus. Below, we focus on unraveling the molecular mechanism by which MrrA impacts the general stress response pathway.

### MrrA is a central phosphorylation hub for multiple histidine kinases.

**FIG 2:**
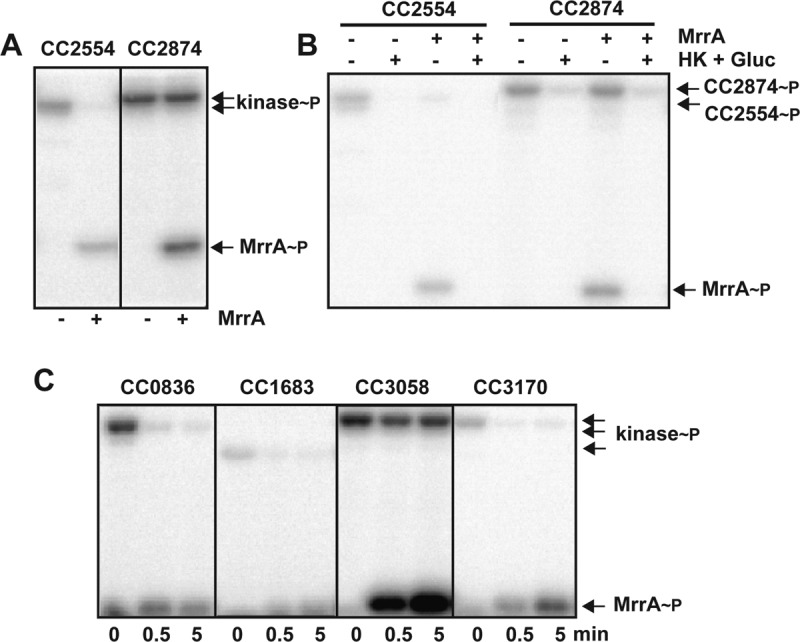
MrrA is phosphorylated by several upstream kinases. (A) Autophosphorylation of histidine kinases CC2554 (left) and CC2874 (right) and phosphotransfer to MrrA. Kinases (5 µM) and MrrA (10 µM) were mixed with 500 µM ATP and 2.5 µCi of [γ-^32^P]ATP (3,000 Ci mmol^−1^) as indicated. Reactions were carried out for 15 min at room temperature, and reaction mixtures were analyzed by SDS-PAGE and autoradiography. The positions of phosphorylated proteins on the gel are indicated on the right. (B) ATP depletion results in back-transfer of phosphate from MrrA to CC2554 (left) and CC2874 (right). Kinase reaction mixtures were incubated with and without MrrA as indicated in panel A for 15 min. ATP was depleted by adding hexokinase (1.5 U) and d-glucose (5 mM). The positions of phosphorylated proteins on the gel are indicated on the right. (C) Several histidine kinases of the HWE subfamily transfer phosphate to MrrA. Purified kinases CC0836, CC1683, CC3058, and CC3170 were mixed with ATP as indicated in panel A, and autophosphorylation reactions were carried out for 30 min. MrrA was then added to the reaction mixtures, and samples were taken at the time points indicated. The positions of phosphorylated proteins on the gel are indicated on the right.

SDRRs function in phosphotransfer reactions or as allosteric regulators via protein-protein interaction. To identify MrrA interaction partners or phosphodonors, we used yeast two-hybrid screening and coimmunoprecipitation. These approaches identified several candidate proteins, including the three histidine kinases CC2501, CC2554, and CC2874 (Table S1). *In vitro* phosphorylation assays with purified proteins failed to show phosphotransfer from CC2501 to MrrA (data not shown) but demonstrated rapid transfer from CC2554 and CC2874 to the conserved Asp53 residue of MrrA (Fig. 2A and S2A). Moreover, when ATP was depleted upon addition of hexokinase and glucose (33), MrrA was efficiently dephosphorylated (Fig. 2B). Although CC2874 harbors a C-terminal Rec domain, this part of the kinase is not required for autophosphorylation and phosphotransfer to MrrA (Fig. S2A). These experiments demonstrated that CC2874 and CC2554 are cognate histidine kinases of MrrA and that both enzymes are able to dephosphorylate MrrA upon ATP depletion.

10.1128/mBio.00809-18.2 FIG S2  (A) Asp53 is the phosphoacceptor residue of MrrA. Autophosphorylation of histidine kinases CC2874 and CC2874 lacking its C-terminal receiver domain (ΔREC) and phosphotransfer to MrrA were analyzed in the presence of 500 µM ATP and 2.5 µCi [γ-^32^P]ATP (3,000 Ci mmol^−1^). MrrA wild type or D53N mutant was used as indicated. Reactions were carried out for 15 min at room temperature and analyzed by SDS-PAGE and autoradiography. The positions of phosphorylated proteins on the gel are indicated on the right. (B) Alignment of all C. crescentus HWE and HisKA2 histidine kinases based on the presence of the highly conserved HRXXN motif in their DHp domains (34). MUSCLE alignments were performed in Geneious v7.1.7 (Biomatters Ltd., Auckland, New Zealand). (C) Autophosphorylation of nine C. crescentus HWE histidine kinases (upper panel) and phosphotransfer to MrrA (lower panel). Phosphorylation reactions were carried out as indicated in panel A. (D) Schematic representation of the domain organization of the C. crescentus HWE/HisKA2 subfamily of histidine kinases. Highlighted with a red asterisk is the only HisKA kinase that phosphorylates MrrA. Graphics were generated by SMART (http://smart.embl-heidelberg.de). Download FIG S2, PDF file, 2.1 MB. Copyright © 2018 Lori et al. 2018 Lori et al. This content is distributed under the terms of the Creative Commons Attribution 4.0 International license.

A recent study had implicated the SDRR SdrG in the general stress response of the alphaproteobacterium Sphingomonas melonis Fr1 (34). Sequence comparison revealed that SdrG and MrrA harbor a conserved PFXFATG(G/Y) motif that distinguishes these proteins from prototypical response regulators (35). Moreover, SdrG and MrrA are best bidirectional hits in BLAST searches, indicating that SdrG and MrrA are orthologs. SdrG is phosphorylated by most members of the HisKA2 and HWE subfamilies of histidine kinases in S. melonis (34). Likewise, CC2554 is a member of the HWE subfamily, while PhyK and LovK, two proteins that have previously been implicated in the general stress response pathway in C. crescentus, belong to the HisKA2 subfamily (Fig. S2B) (25, 29). This prompted us to test if other HisKA2/HWE kinases of C. crescentus are able to phosphorylate MrrA. LovK, PhyK, and the remaining nine HWE/HisKA2 kinases of C. crescentus (CC0629, CC0836, CC1683, CC2909, CC3048, CC3058, CC3170, CC3198, and CC3569) (Fig. S2B) were purified and analyzed for autophosphorylation and phosphotransfer to MrrA. Of the 11 kinases tested, five showed robust autophosphorylation under the conditions used. Four of the five kinases that were active *in vitro* showed rapid phosphotransfer to MrrA (Fig. 2C and S2C). Notably, among the kinases lacking autokinase activity were also PhyK and LovK (see below).

Altogether, these results demonstrated that MrrA is phosphorylated by multiple HWE and HisKA2 histidine kinases. Kinase CC2874, a classical HisKA histidine kinase that does not belong to the HWE or HisKA2 subfamilies, also efficiently phosphorylated MrrA. These data established MrrA as a central phosphorylation hub that collects phosphate from members of different kinase families to control C. crescentus general stress response activity.

### MrrA controls the activation of the general stress response proteins PhyK and LovK.

**FIG 3:**
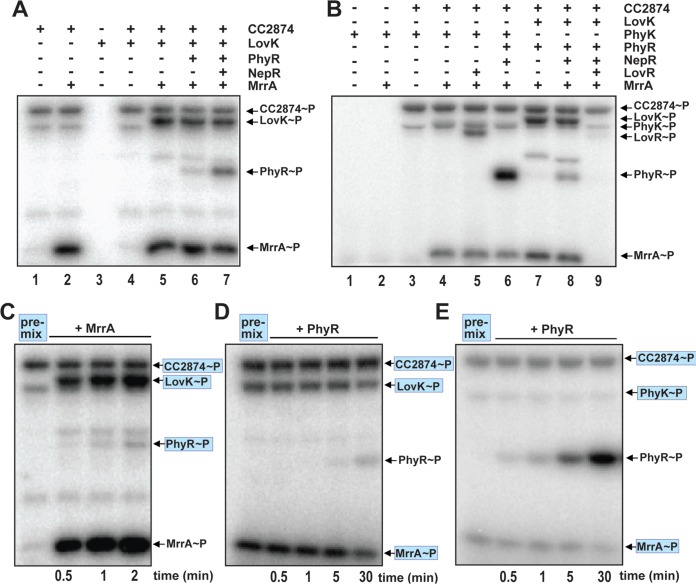
MrrA phosphorylates components of the general stress response. (A) MrrA transfers phosphate to LovK and PhyR. Five hundred micromoles ATP and 2.5 µCi [γ-^32^P]ATP (3,000 Ci mmol^−1^) were mixed with the proteins indicated, reactions were carried out for 15 min at room temperature, and reaction mixtures were analyzed by SDS-PAGE and autoradiography. Note that LovK does not display autokinase activity by itself but is readily phosphorylated if MrrA is present. The positions of phosphorylated proteins on the gels are indicated on the right. (B) MrrA transfers phosphate to PhyK and PhyR. Phosphorylation reactions were assembled and run as in panel A. The results obtained for PhyK were similar to the results obtained for LovK. PhyK does not display autokinase activity but is readily phosphorylated if MrrA is present. Note that the MBP-CC2874 preparation contains a fraction of cleaved protein that represents CC2874 without MBP tag, such that autophosphorylation of (MBP-)CC2874 yields two radiolabeled bands (lane 3), the lower one of which migrates only slightly faster than the PhyK band. The positions of phosphorylated proteins on the gels are indicated on the right. (C) Phosphorylation of MrrA and LovK is rapid, while phosphorylation of PhyR is slow. Phosphorylation reaction mixtures with radiolabeled ATP and purified CC2874, LovK, PhyR, and NepR were preincubated for 30 min (premix). Purified MrrA was then added to the reaction mixtures, and samples were taken at the time points indicated. The positions of phosphorylated proteins on the gels are indicated on the right with proteins present in the premix being highlighted in blue (C to E). (D) PhyR phosphorylation through LovK is slow and inefficient. Phosphorylation reaction mixtures with radiolabeled ATP and purified CC2874, LovK, MrrA, and NepR were preincubated for 30 min (premix). Purified PhyR was then added to the reaction mixtures, and samples were taken at the time points indicated. (E) PhyR phosphorylation through PhyK is rapid and efficient. Phosphorylation reaction mixtures with radiolabeled ATP and purified CC2874, PhyK, MrrA, and NepR were preincubated for 30 min (premix). PhyR was then added to the reaction mixtures, and samples were taken at the time points indicated.

Previous studies demonstrated that the histidine kinase PhyK is essential for the general stress response in C. crescentus *in vivo* (25, 29). In contrast, LovK was proposed to be a negative regulator of the general stress response by promoting dephosphorylation of PhyR (25) (Fig. 1A). However, biochemical evidence for the catalytic activity of PhyK as a genuine histidine kinase and for a role of LovK and PhyK in PhyR (de)phosphorylation is missing. Since the above genetic studies identified MrrA as a positive regulator of the general stress response, we wondered whether MrrA could act as a direct activator of PhyK or LovK. *In vitro* phosphorylation experiments revealed that PhyK and LovK were readily phosphorylated in the presence of CC2874, MrrA, and ATP but not when incubated with ATP alone (Fig. 3A, compare lanes 3 and 5; Fig. 3B, compare lanes 1 and 4), with phosphotransfer to LovK occurring within seconds (Fig. 3C). Next, we tested if LovK and PhyK could pass on phosphoryl groups to PhyR. Because it was previously shown that PhyR of S. melonis was efficiently phosphorylated by cognate histidine kinases only in the presence of the anti-sigma factor NepR, NepR was included in the phosphotransfer reaction mixtures containing PhyR (34). Indeed, inclusion of NepR in phosphotransfer reactions to PhyR strongly enhanced PhyR phosphorylation (Fig. 3A, compare lanes 6 and 7). Although both PhyK and LovK were able to phosphorylate PhyR under these conditions (Fig. 3A and B), time course experiments revealed that phosphorylation by PhyK was rapid and efficient (Fig. 3E), while phosphorylation by LovK was slow and comparably weak (Fig. 3D). These results are in line with the notion that PhyK, but not LovK, is the primary phosphodonor for PhyR (Fig. 1A).

**FIG 4:**
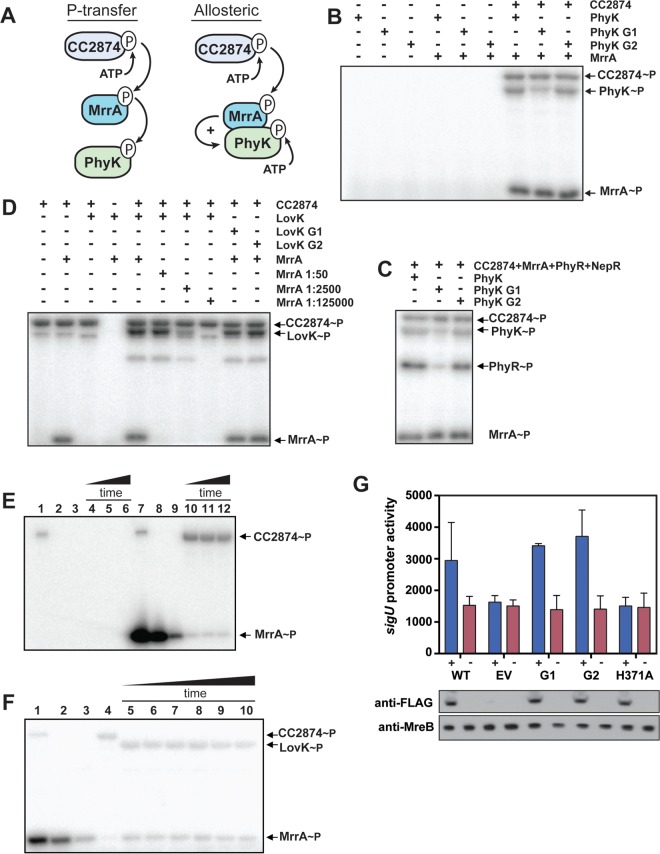
LovK and PhyK are phosphotransferases that are activated by MrrA~P. (A) Schematic representation of two possible modes of action of MrrA~P, phosphotransfer to and allosteric activation of PhyK and LovK. For reasons of simplicity, only PhyK is shown. (B) PhyK phosphorylation does not require a conserved CA domain. Phosphorylation reactions with radiolabeled ATP, the kinase CC2874, MrrA, and different PhyK variants. Purified PhyK wild type or mutant variants harboring mutations in the G1 (G514A/G516A) or G2 (G526A) box of the ATP-binding site were used as indicated. The positions of phosphorylated proteins on the gel are indicated on the right. (C) LovK phosphorylation does not require a conserved CA domain. Phosphorylation reactions with radiolabeled ATP, kinase CC2874, MrrA, and different LovK variants. Purified LovK wild type or mutant variants harboring mutations in the G1 (G319A/G321A) or G2 (G332A) box ATP-binding site of the CA domain were used as indicated. MrrA dilution factors are indicated in each lane. The positions of phosphorylated proteins on the gel are indicated on the right. (D) Phosphotransfer to PhyR does not require a conserved CA domain. Phosphorylation reaction mixtures containing radiolabeled ATP, kinase CC2874, MrrA, PhyR, NepR, and different PhyK variants are as in panel C. The positions of phosphorylated proteins on the gel are indicated on the right. (E) Preparation of purified MrrA~P. Kinase CC2874 alone or with MrrA was phosphorylated with radiolabeled ATP (lanes 1 and 7), and CC2874 was subsequently removed using anti-MBP magnetic beads (lanes 2 and 8). Next, ATP was hydrolyzed by treating mixtures with hexokinase and glucose (lanes 3 and 9). CC2874 was added back to ATP-depleted samples, and mixtures were incubated for 0.5, 1.0, and 5 min (lanes 4 to 6 and 10 to 12). (F) Purified MrrA~P transfers phosphate to LovK. MrrA~P was prepared as in panel E (lane 1), CC2874 was removed (lane 2), and ATP was degraded (lane 3). Fresh CC2874 (lane 4) or LovK was added, and phosphotransfer from MrrA~P was monitored after 10 s, 20 s, 1 min, 2 min, 10 min, and 20 min (lanes 5 to 10, respectively). (G) ATP binding is not required for PhyK activity in the general stress response. Δ*lovK* strains harboring an empty vector (EV) or a plasmid expressing different *phyK* alleles from a cumate-inducible promoter were analyzed. Plasmid-driven variants of PhyK contained mutations in the G1 or G2 box of the ATP-binding pocket (see above) or in the conserved phosphoacceptor His371. SigT-dependent *sigU* promoter activity (Miller units) was determined using a *lacZ* promoter fusion in strains grown in the presence (+) or absence (−) of cumate. PhyK variants harbored a C-terminal 3×FLAG tag that allowed monitoring their expression by immunoblot analysis (lower panels). An immunoblot with anti-MreB antibodies is shown as a control. Note that the *sigUp-lacZ* reporter fusion used in these experiments differed from the one used in experiments above (Fig. 1D) and shows higher basal activity (compare wild-type PhyK and empty-vector control in panel G).

The data above suggested that phosphorylated MrrA acts as a direct activator of PhyK and LovK, explaining the strong stress response phenotype observed for the Δ*mrrA* strain *in vivo*. We reasoned that MrrA~P could either allosterically activate PhyK and LovK kinase activities (10) or serve as a phosphodonor for PhyK and LovK (Fig. 4A). The latter scenario would imply that PhyK and LovK do not serve as kinases but have adopted a role as histidine phosphotransferases (HPts). In fact, DHp (dimerization and histidine phosphotransfer) domains of histidine kinases and HPt domains are structurally very similar (36–38). To distinguish between the two possibilities, we purified variants of PhyK harboring mutations in conserved residues of the G1 or G2 boxes of its catalytic (CA) domain that are essential for ATP binding. If PhyK is a bona fide kinase that is allosterically activated by MrrA~P, mutations in the ATP-binding pocket should abolish autophosphorylation (6). However, not only did both mutant proteins still accumulate radiolabel in a CC2874- and MrrA-dependent manner (Fig. 4B), but they also were able to phosphorylate PhyR (Fig. 4C). Together, this argued that PhyK serves as a phosphotransferase to shuttle phosphate from MrrA to PhyR. Similarly, LovK G1 and G2 mutant variants were phosphorylated indistinguishably from wild-type LovK in a reaction that required the kinase CC2874 and MrrA (Fig. 4D).

To corroborate the idea that LovK and PhyK act not as kinases but rather as HPt-like proteins, we established a procedure to purify radiolabeled MrrA~P to directly follow phosphotransfer from MrrA to LovK or PhyK in the absence of ATP. To this end, MrrA was phosphorylated *in vitro* using MBP-tagged (maltose binding protein) CC2874 and radiolabeled ATP (Fig. 4E, lane 7). MBP-CC2874 was then removed from the reaction mix with anti-MBP magnetic beads (Fig. 4E, lane 8), and the remaining ATP was depleted from the MrrA~P preparation using hexokinase and glucose (Fig. 4E, lane 9). As a control, the same procedure was performed without MrrA (Fig. 4E, lanes 1 to 3). When CC2874 was added to this mock preparation, it failed to accumulate radiolabel, indicating that ATP was efficiently removed (Fig. 4E, lanes 4 to 6). In contrast, CC2874 was readily phosphorylated when incubated with the preparation containing MrrA~P, indicating back-transfer from MrrA~P to CC2874 (Fig. 4E, lanes 10 to 12). Similarly, when isolated MrrA~P was incubated with LovK or PhyK, rapid accumulation of radiolabel on both proteins was observed (Fig. 4F, compare lane 3 to lanes 5 to 10; see also Fig. S3A). No radiolabel accumulated on LovK or PhyK when incubated with cold, i.e., nonradiolabeled, MrrA~P, even though radiolabeled ATP was present in the reaction mixture (Fig. S3B). Together, these results strongly implied that MrrA acts as a shuttle to transfer phosphate from the kinase CC2874 to LovK and PhyK. These experiments also suggested that LovK and PhyK do not primarily act as kinases but rather serve as phosphotransfer proteins to control the activity of PhyR and, ultimately, that of SigT.

10.1128/mBio.00809-18.3 FIG S3  MrrA~P efficiently transfers phosphate to LovK and PhyK. (A) Phosphorylation reactions with purified CC2874 and MrrA were carried out as described for Fig. 4F. The CC2874 kinase was then purified away using anti-MBP magnetic beads (lane 2). Remaining ATP was degraded using glucose and hexokinase (lane 3). Purified LovK was added with [γ-^32^P]ATP, and accumulation of LovK~P was monitored after 10 s, 20 s, 1 min, 2 min, 10 min, and 20 min (lanes 5). Reactions were analyzed by SDS-PAGE and autoradiography. The positions of phosphorylated proteins on the gel are indicated on the right. (B) Phosphorylation reactions as in panel A, but MrrA was initially phosphorylated by CC2874 in the presence of unlabeled ATP (lane 1). Samples were then treated as in panel A (lanes 2 to 4), and purified LovK (upper panel) or PhyK (lower panel) was added together with [γ-^32^P]ATP. Phosphorylation of LovK and PhyK was monitored over time as in panel A. Download FIG S3, PDF file, 0.7 MB. Copyright © 2018 Lori et al. 2018 Lori et al. This content is distributed under the terms of the Creative Commons Attribution 4.0 International license.

To test the physiological relevance of our biochemical data, we sought to analyze general stress response activity of C. crescentus strains harboring mutations in the G1 and G2 boxes of PhyK. To this end, 3×FLAG-tagged mutant variants of PhyK were expressed in *trans* from a cumate-inducible promoter in a Δ*phyK* strain and SigT activity was monitored using a *sigU*-*lacZ* reporter. In line with our biochemical data, both PhyK mutants fully complement the Δ*phyK* phenotype (Fig. 4G). In contrast, PhyK with a mutation of the conserved phosphoaccepting histidine (H371A) failed to rescue SigT activity. Thus, ATP-binding and autokinase activity are not required for PhyK activity *in vivo*, strengthening the notion that it acts exclusively as a phosphotransfer protein to promote PhyR phosphorylation.

### LovR is a selective phosphate sink for MrrA but not for PhyR.

**FIG 5:**
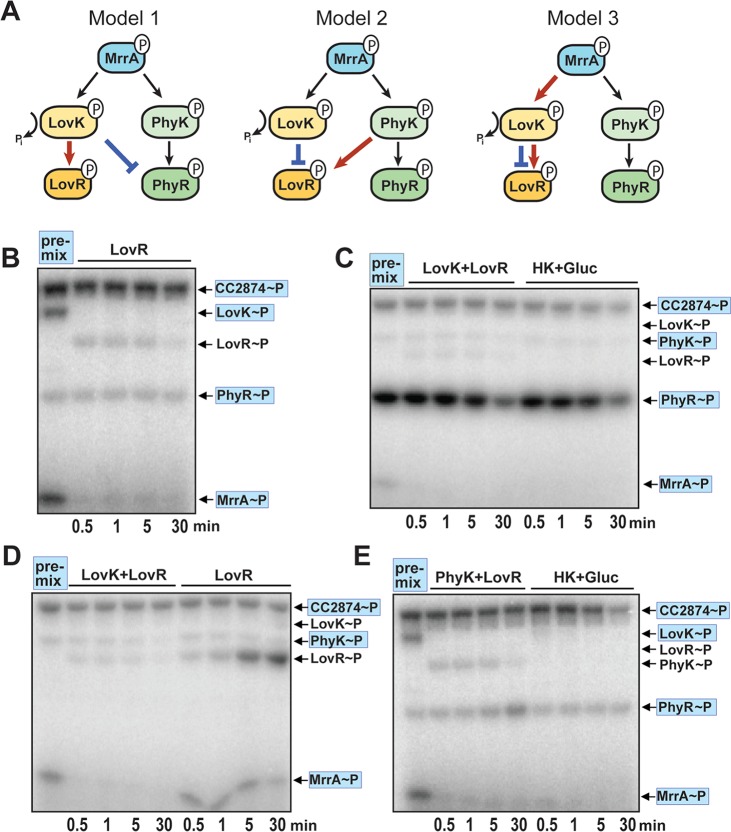
LovK and LovR rapidly dephosphorylate MrrA but not PhyK or PhyR. (A) Models for the role of LovK and LovR in the regulation of the C. crescentus general stress response. PhyR activity could be reduced by LovK acting as phosphatase for PhyR~P with LovR as terminal phosphate sink (model 1). PhyK activity could be reduced by direct phosphotransfer to LovR with LovK acting as phosphatase for LovR~P (model 2). MrrA activity could be reduced by phosphotransfer to LovK and LovR. In this scenario, LovR serves as terminal phosphoacceptor with LovK serving both as HPt and as phosphatase for LovR (model 3). (B) LovK is not a phosphatase for PhyR. Phosphorylation reaction mixtures with radiolabeled ATP and purified CC2874, LovK, PhyR, MrrA, and NepR were incubated for 30 min (premix). Phosphorylation levels of all proteins were monitored before and after addition of LovR at time points indicated. The positions of phosphorylated proteins on the gels are indicated on the right with proteins present in the premix being highlighted in blue (B to E). (C) PhyK does not transfer phosphate to LovR and LovK. Phosphorylation reaction mixtures with radiolabeled ATP and purified CC2874, MrrA, PhyR, NepR, and PhyK were incubated for 30 min (premix). Phosphorylation levels of all proteins were monitored before and after the addition of LovK/LovR or after addition of hexokinase and glucose at time points indicated. Note that the first lane in the autoradiograph is identical to the last lane in the autoradiograph shown in Fig. 3E. (D) LovR~P accumulates in the absence of LovK. Phosphorylation reaction mixtures with radiolabeled ATP and purified CC2874, MrrA, and PhyK were incubated for 30 min (premix). Phosphorylation levels of all proteins were monitored before and after addition of LovK and LovR or after addition of LovR alone at time points indicated. (E) PhyK does not transfer phosphate to LovR and LovK. Phosphorylation reaction mixtures with radiolabeled ATP and purified CC2874, MrrA, LovK, PhyR, and NepR were incubated for 30 min (premix). Phosphorylation levels of all proteins were monitored before and after the addition of PhyK and LovR or glucose/hexokinase at the time points indicated. Note that the first lane in the autoradiograph is identical to the last lane in the autoradiograph shown in Fig. 3D.

In the experiments described above, we established that MrrA samples information from multiple upstream kinases and, in response, shuttles phosphoryl groups to both LovK and PhyK. Whereas these observations are in good agreement with the genetic data demonstrating that MrrA and PhyK are part of the C. crescentus general stress response, they do not account for the described negative effect of LovK on the general stress response (25). Strains lacking LovK or LovR showed increased SigT activity and SigT-dependent survival under stress conditions; moreover, cooverexpression of *lovK* and *lovR*, but not of *lovK* or *lovR* alone, abolished SigT activity (25). To explain these genetic results and to rationalize how LovK and LovR downregulate SigT activity, it was proposed that LovK, together with LovR, could serve to promote PhyR dephosphorylation. Such a mechanism could be based on (i) LovK serving as a phosphatase of PhyR with LovR as the terminal phosphate sink (Fig. 5A, model 1) or on (ii) phosphotransfer from PhyK to LovR with LovK acting as a LovR phosphatase (Fig. 5A, model 2) (25). Considering our findings that MrrA is positioned upstream of PhyK and LovK, we reasoned that the role of LovK and LovR may be to drain phosphate away from the PhyK-PhyR branch by rerouting phosphate flux via MrrA (Fig. 5A, model 3).

When PhyR was phosphorylated in the presence of CC2874, MrrA, and LovK, addition of LovR resulted in the instant loss of LovK~P and MrrA~P but not PhyR~P (Fig. 5B, compare lane 1 to lanes 2 to 5). Notably, under these conditions only weak accumulation of LovR~P was observed, arguing that LovR~P is subject to rapid dephosphorylation. Thus, LovK does not serve as a phosphatase for or phosphoacceptor of PhyR~P *in vitro*. To test if PhyR was dephosphorylated via PhyK and LovR, PhyR was phosphorylated in the presence of CC2874, MrrA, and PhyK, and LovK and LovR were added to the reaction mixture. This led to an instant loss of MrrA~P but not of PhyR~P or PhyK~P (Fig. 5C, compare lane 1 to lanes 2 to 5). Similarly, MrrA~P was depleted upon addition of LovK and LovR to a reaction mix containing CC2874, MrrA, and PhyK (Fig. 5D, compare lane 1 to lanes 2 to 5). In contrast, when LovR alone was added to a premix containing CC2874, MrrA, and PhyK, MrrA~P was not depleted and instead LovR~P accumulated (Fig. 5D, compare lane 1 to lanes 6 to 9). These observations support the idea that LovK and LovR function together to drain phosphoryl groups from MrrA and that LovR~P does not undergo spontaneous dephosphorylation but that LovK acts as a phosphatase for LovR~P. Similarly, when PhyR was phosphorylated in the presence of CC2874, MrrA, and LovK, addition of PhyK and LovR led to a rapid loss of MrrA~P and LovK~P but not of PhyR~P (Fig. 5E, compare lane 1 to lanes 2 to 5). Finally, we tested if PhyR~P could be dephosphorylated upon depletion of ATP by hexokinase treatment and the resulting switching of CC2874 into phosphatase mode. Irrespective of how PhyR was phosphorylated, both PhyR and PhyK retained the radiolabel, while MrrA~P and LovK~P were rapidly dephosphorylated under these conditions (Fig. 5C and E, compare lanes 1 to lanes 6 to 9).

In conclusion, these results strongly argue against models 1 and 2 in Fig. 5A but support model 3, in which the negative effect of LovK and LovR on the general stress response is a direct result of draining phosphate from MrrA. In contrast, cross-phosphorylation reactions between the LovKR and the PhyKR branch are unlikely. Importantly, rather than acting as a prototypical phosphatase of MrrA~P, LovK seems to act as a phosphotransferase to shuttle phosphoryl groups from MrrA to LovR. In a final step, LovK then acts as a genuine phosphatase for LovR~P, a function that is essential to make LovKR an efficient phosphate sink.

## DISCUSSION

**FIG 6:**
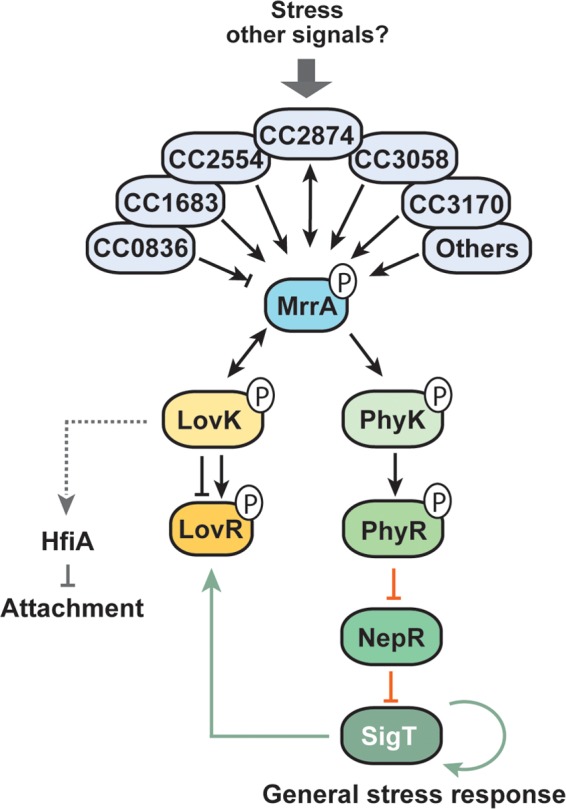
Model of MrrA function in the general stress response and developmental control of C. crescentus. We propose that members of different subfamilies of histidine kinases sample different forms of stress and possibly other signals. Signaling converges through the phosphorylation of the single-domain response regulator MrrA. MrrA~P divergently distributes phosphate to the phosphotransfer proteins LovK and PhyK. Our data imply that PhyK~P is the principal phosphodonor for PhyR to activate the general stress response. LovK and LovR form a potent phospho-sink that involves LovK phosphotransfer to LovR, followed by rapid LovK-mediated removal of phosphoryl groups from LovR~P. Because *lovK* and *lovR* expression is SigT controlled, these proteins may constitute a feedback control mechanism that helps to adapt an excessive stress response. Alternatively, signals sensed by LovK may alter LovKR activity by rerouting phosphate flux from MrrA and fine-tune PhyR and ultimately SigT activity. Additional cellular processes like holdfast formation and surface attachment are integrated with the MrrA phosphorylation cascade through LovK. Green, black, and red arrows/bars indicate transcriptional regulation, phosphorylation, and inhibitory protein-protein interactions, respectively. Gray arrows indicate regulatory links for which the mechanistic details are unknown. For simplicity, stimulation of PhyR phosphorylation by NepR and the positive effect of SigT on *nepR*, *phyR*, *phyK*, and *lovK* expression are not shown.

Single-domain response regulators were previously shown to play important roles in C. crescentus cell cycle progression, development, and behavior (15, 18, 23, 39). In the present study, we describe a novel single-domain response regulator in C. crescentus, MrrA, that is involved in a range of physiological processes, including growth, motility, and attachment. Surprisingly, MrrA is also an integral and essential component of the general stress response in this organism. We propose a model where MrrA is phosphorylated by several histidine kinases of different subclasses that are involved in the perception of diverse stress factors (25, 29, 34, 40). Once phosphorylated, MrrA shuttles phosphoryl groups to PhyK, which in turn phosphorylates PhyR, thereby inducing the partner switch triggering the general stress response (Fig. 6). However, MrrA can also shuttle phosphate to LovK, a protein that, together with its cognate response regulator LovR, acts as a negative regulator of the general stress response. By draining phosphoryl groups from MrrA~P, LovK and LovR limit phosphorylation of PhyK and thus downregulate the general stress response. In addition, MrrA controls attachment by modulating the expression of *hfiA*, a process that is dependent on LovK, but the mechanistic details of this control are currently unclear. Altogether, this puts MrrA at the center of a complex signal transduction cascade that coordinates the C. crescentus stress response with behavioral adaptations.

### Multiple upstream histidine kinases phosphorylate MrrA.

The observation that multiple histidine kinases of the HWE and HisKA2 subclasses phosphorylate MrrA suggests that multiple signals are integrated at the level of MrrA phosphorylation. This notion seems reasonable since the general stress response is thought to respond to and protect from several unrelated stresses. In fact, in the related alphaproteobacterium S. melonis, most HWE and HisKA2 kinases converge on PhyR phosphorylation with different kinases sensing different stresses. Interestingly, deletion of these kinases does not fully abrogate general stress response activity, suggesting that kinases outside the HWE and HisKA2 subclasses also contribute to stress response activation (34). This is in agreement with our observation that MrrA is efficiently phosphorylated by the kinase CC2874, which belongs to the HisKA subfamily. We speculate that the number of kinases that can phosphorylate MrrA is even larger, since several HWE/HisKA2 kinases failed to autophosphorylate *in vitro* and because HisKA kinases were not systematically tested for phosphotransfer to MrrA.

The molecular cues that are sensed by the histidine kinases upstream of MrrA are currently unknown. Most kinases harbor classical sensing domains such as PAS and GAF domains, suggesting that they can sense stresses directly (see Fig. S2D in the supplemental material). Although few kinases have been assigned specific sensory functions in the general stress response of alphaproteobacteria, LOV domains sensing blue light have been linked to this pathway in several organisms (25, 26, 28, 34, 41–45). LovK harbors a LOV domain and was previously shown to respond to blue light *in vitro* and to mediate attachment dependent on this stimulus (23). It is possible that phosphate flux through MrrA is modulated by blue light. In addition, the LOV domain of LovK was proposed to respond to redox conditions (25). This is based on the findings that a strain lacking PhyK and LovR but overproducing LovK still responds to oxidative stress. Although we cannot rule out this possibility, our data offer an alternative explanation for this observation, namely, that an upstream kinase of MrrA responds to redox conditions and that LovK in this specific genetic context simply serves as an HPt shuttling phosphoryl groups to PhyR. A future challenge will be to assign sensory functions to individual kinases involved in the general stress response.

### PhyK is a phosphotransfer protein.

Previous studies from independent groups have identified PhyK as a central component of the general stress response. It was assumed that PhyK acts as a histidine kinase, which, in response to specific stimuli, phosphorylates the PhyR response regulator (25, 29). This assumption seemed plausible since PhyK has a conserved CA domain and harbors a putative periplasmic sensing domain. However, PhyK lacks autophosphorylation activity and rather functions as an HPt, accepting phosphate directly from MrrA~P. Moreover, conserved residues for ATP binding were dispensable for PhyK function *in vivo*. Finally, a Δ*mrrA* mutant quantitatively phenocopies a Δ*sigT* or Δ*phyK* strain in terms of its defect in general stress response activation, suggesting that in an Δ*mrrA* strain PhyK lacks residual kinase activity. It is currently unclear why the CA domain of PhyK (and LovK) is so well conserved despite its apparent lack of enzymatic activity. It is possible that the CA domain plays an allosteric role in signal transduction, contributes to structural stability and integrity, or serves scaffolding functions. Of note, the S. melonis ortholog of PhyK, PhyP, harbors a degenerate CA domain and was proposed to act as a phosphatase of PhyR (46, 47). Whether PhyP also shuttles phosphoryl groups between PhyR and the MrrA ortholog SdrG in this organism is unknown. We also cannot rule out that PhyK has kinase activity under specific conditions, under which it is able to directly respond to particular stresses. A previous study identified a cysteine residue in the periplasmic domain of PhyK that, when mutated, abolished PhyK function, suggesting that it is involved in stress sensing (29). If so, PhyK would be able to integrate multiple signals through its periplasmic sensor domain and through phosphorylation by MrrA~P. In fact, an earlier study reported autophosphorylation of LovK *in vitro* (23), although with our LovK expression construct and under the experimental conditions employed, we do not observe a significant degree of LovK autophosphorylation. Importantly, our *in vivo* experiments clearly demonstrate that autokinase activity of PhyK is dispensable for PhyK function, strongly arguing that its essential function in the general stress response is that of a histidine phosphotransferase.

### Role of LovK and LovR as inhibitors of the general stress response.

Recently, LovK and LovR were described as negative regulators of the general stress response (25). Different models were proposed for LovKR control that postulated dephosphorylation of PhyR as the mechanism to shut down the general stress response, predicting direct cross talk between the PhyKR and LovKR branches (25). While our data confirmed that LovR can rapidly deplete phosphate from LovK, we did not observe dephosphorylation of PhyR~P or PhyK~P *in vitro*. Thus, the LovKR and PhyKR branches do not seem to cross-talk directly. Rather, LovKR restricts phosphate flow toward PhyKR by draining phosphoryl groups from the shared upstream component MrrA. Because NepR was always present during *in vitro* reactions, we cannot exclude that PhyR alone can be dephosphorylated by LovK or PhyK. However, this seems unlikely given the recent observation that NepR binding and PhyR phosphorylation are cooperative (48, 49). Since the expression of *lovKR* is positively controlled by SigT, LovKR likely constitute a negative-feedback loop that serves to dampen the stress response (Fig. 6). Our findings that LovK and LovR do not directly lead to PhyK or PhyR dephosphorylation imply that the dampening effect on SigT activity is provided by diverting phosphoryl groups away from MrrA and thus restricting future phosphorylation of PhyR. In combination with the stoichiometric upregulation of the general stress response core components PhyR, NepR, and SigT, this would ultimately result in the accumulation of unphosphorylated PhyR and the shutdown of the response (26, 28).

Our data show that MrrA~P can be dephosphorylated by at least two of its upstream kinases, CC2554 and CC2874, upon depletion of ATP (Fig. 2B). Moreover, CC2874 directly accepts phosphoryl groups from MrrA~P (Fig. 4E). We speculate that upstream components of MrrA are generally able to switch into phosphatase mode and shut down the response, possibly upon cessation of their respective input signals. Thus, the extent of MrrA phosphorylation may simply be dictated by mass action, i.e., free phosphate flow between up- and downstream kinases and MrrA, which in turn depends on whether kinases are in kinase or phosphatase mode. We have not quantitatively assessed MrrA autodephosphorylation in this study. However, based on the fact that we could easily isolate stable MrrA~P *in vitro* (Fig. 4E), we propose that this process plays a minor role in phosphorelay dynamics. This is in good agreement with the presence of conserved Asn and Tyr residues at the D+2 and T+2 positions (50).

### Conserved and divergent roles of MrrA in alphaproteobacteria.

In this study, we show that MrrA is an essential and central component of the general stress response in C. crescentus. In contrast to prototypical response regulators, MrrA lacks one of the residues involved in the Y-T coupling mechanism required for intramolecular signal transduction of Rec domains. Instead, it harbors the recently described FATGUY motif (35, 51, 52). Three other FATGUY response regulators are described, SdrG of S. melonis, Mext_0407 of Methylobacterium extorquens, and Sma0114 of Sinorhizobium meliloti (34, 53, 54). While the first two proteins are involved in the general stress response of these organisms, the latter is involved in succinate-mediated catabolite repression and polyhydroxybutyrate production; whether or not Sma0114 also plays a role in the general stress response has not been tested. Hence, it seems reasonable to propose that MrrA and other members of this subfamily of response regulators play a conserved role in the general stress response of alphaproteobacteria.

The degree to which MrrA orthologs contribute to general stress response activity seems to differ between organisms. In S. melonis and M. extorquens, deletion of the *mrrA* orthologs *sdrG* and Mext_0407 reduces, but does not completely abolish, general stress response activity (34, 53). In S. melonis, overexpression of most HWE/HisKA2 kinases leads to the induction of the general stress response. However, only a subset of these kinases require SdrG for this induction (34). Interestingly, in S. melonis, most HWE/HisKA2 kinases phosphorylate both PhyR and SdrG *in vitro*, arguing that specificity determinants of the receiver domains of PhyR and SdrG are similar (34, 35). These observations argue that the regulatory wiring of FATGUY response regulators to up- and downstream histidine kinases is plastic, similar to the plasticity and modularity of the sensory capacities of general stress response kinases themselves, likely reflecting species-specific niche adaptation (26, 28). In addition, FATGUY response regulators seem highly promiscuous with respect to their cognate kinases. This is in contrast to the vast majority of two-component systems that are thought to have evolved toward insulation (55, 56). Because known specificity determinants for histidine kinase-response regulator interaction were identified only for the HisKA subfamily and their prototypical response regulators employing Y-T coupling (57, 58), the molecular details of this promiscuity remain unknown. However, FATGUY response regulators like MrrA may have evolved as central phosphorylation hubs to integrate the phosphorylation status of multiple two-component systems, thereby coordinating the general stress response with cellular behavior and development. Coimmunoprecipitation experiments with MrrA (Table S1A) also identified the diguanylate cyclase DgcB (59, 60) and ChpT, a histidine phosphotransferase that plays a central role in cell cycle progression (18). It is thus possible that MrrA also intersects with c-di-GMP signaling and cell cycle control.

Networks combining multiple inputs with multiple output processes through a central “knot” are termed “bow-tie.” Bow-tie architectures in metabolism or signal transduction were proposed to facilitate independent evolution of the input and output functions without affecting the regulatory core (61) and also compress cellular input information (62). One of the primary challenges for bacteria expanding their ecologic niches is to rapidly evolve adaptation to a plethora of novel stresses encountered at new sites and to effectively link this information with existing or emerging processes of stress response and behavior. We propose that the bow-tie architecture of the phosphorylation network controlling stress response and behavior in C. crescentus ultimately facilitates niche adaptation.

## MATERIALS AND METHODS

### Oligonucleotides, plasmids, strains, and media.

Oligonucleotides, plasmids, and bacterial strains are listed in Table S2 in the supplemental material. C. crescentus was grown in PYE medium or minimal medium with glucose (M2G) at 30°C (63). E. coli DH5α was used as a host for cloning and grown in LB at 37°C. When required, the growth media were supplemented with antibiotics at the following concentrations (liquid/solid medium): for C. crescentus, 5/50 µg ml^−1^ of ampicillin, 5/20 µg ml^−1^ of kanamycin, 2.5/5 µg ml^−1^ of tetracycline, 1/2 µg ml^−1^ of chloramphenicol, and 15/20 µg ml^−1^ of nalidixic acid; for E. coli, 50/100 µg ml^−1^ of ampicillin, 30/50 µg ml^−1^ of kanamycin, 12.5/12.5 µg ml^−1^ of tetracycline, 20/30 µg ml^−1^ of chloramphenicol, and 15/30 µg ml^−1^ of nalidixic acid.

10.1128/mBio.00809-18.5 TABLE S2  Plasmids, strains, and oligonucleotides used in this study. Download TABLE S2, XLSX file, 0.02 MB. Copyright © 2018 Lori et al. 2018 Lori et al. This content is distributed under the terms of the Creative Commons Attribution 4.0 International license.

### Growth experiments.

Independent C. crescentus cultures were diluted to an optical density at 660 nm (OD_660_) of 0.05 in PYE medium. Three technical replicates (165 µl) of each culture were inoculated in 96-well plates, and growth was monitored at 660 nm every 15 min in a Synergy H4 hybrid reader (BioTek) using Gen5 2.00 software (BioTek) at 30°C under shaking conditions (medium-speed, continuous shaking).

### Attachment and motility assays.

Surface attachment of C. crescentus was determined as described previously (64). Motility assays were carried out as described previously (15).

### Hydrogen peroxide stress assays.

The stress assay was adapted from reference 65. Cells were grown overnight in minimal medium with xylose (M2X). Overnight cultures were diluted back to an OD_660_ of 0.05. Cells were grown for 5 h and again diluted back to an OD_660_ of 0.05. Cultures were split, and one culture was exposed to 0.2 mM H_2_O_2_ for 1 h. H_2_O_2_ (fresh bottle) was diluted back from a 10 mM solution. After the stress treatment, cells were serially diluted 1:10 in M2X and spotted on PYE agar plates.

### Determination of c-di-GMP concentrations.

c-di-GMP extraction and quantification were carried out as described previously (60).

### β-Gal assays.

Independent C. crescentus cultures were grown in PYE to an OD_660_ of 0.3 (except for stationary-phase cultures, which were taken directly from an overnight [ON] culture). Two milliliters of culture was pelleted and resuspended in 2 ml fresh Z buffer (0.06 M Na_2_HPO_4_, 0.04 M NaH_2_PO_4_, 0.01 M KCl, 0.001 M MgSO_4_, 0.3% β-mercaptoethanol). One milliliter was mixed with 100 µl of 0.1% SDS and 20 µl chloroform by vortexing for 10 s and was incubated for 15 to 30 min. Three replicates of 200 µl each were transferred to a 96-well plate, 25 µl of fresh ONPG (*o*-nitrophenyl-β-d-galactopyranoside, 4-mg/ml stock) was added, β-galactosidase (β-Gal) activity was measured in an EL800 plate reader (both from BioTek Instruments) over time, and the maximum slope was plotted as increase of OD_405_ corrected for OD_660_ and volume. Alternatively, β-galactosidase measurements were performed as described previously (66) using pAK504 and pAK505 *lacZ* reporter plasmids, either with exponentially growing cultures (*hfiA-lacZ* reporter fusion) or directly on overnight cultures (*sigUp-lacZ* reporter fusion) grown in PYE. Where appropriate, cumate was included in overnight cultures at a concentration of 100 µg/ml to induce gene expression from promoter P_Q5_ (46). pAK504 was constructed by ligation of a blunted AatII/PciI-fragment derived from pUT18 carrying *bla* and the ColE1 *oriV* into the XmaI site of pAK501 (46). To construct plasmid pAK502, part of *lacZ* was PCR amplified from pAK501 using primers 9060 and 9061, and the product was digested with KpnI/DraIII and cloned into pAK501 digested with the same enzymes. The resulting plasmid carries *lacZ* without a ribosome binding site and start codon and allows the construction of translational *lacZ* fusions. pAK505 was derived from pAK502 by subcloning a SacII/EcoRI fragment carrying *bla* and the ColE1 *oriV* from pAK504 in between the same sites of pAK502. Inserts were cloned in pAK504 and pAK505 using KpnI and XbaI restriction sites and primers described in Table S2.

### Proteome analysis.

Independent cultures of C. crescentus were grown to an OD_660_ of 0.3, and 10 ml of cells was pelleted and dissolved in 200 µl cold lysis buffer (8 M urea, 0.1 M ammonium bicarbonate, 0.1% RapiGest). Cells were lysed by ultrasonication (Vial Tweeter; Hielscher) (2 times for 10 s each, amplitude 100, cycle 0.5) and shaking in a Thermomixer C (Eppendorf; 5 min, 1,400 rpm, room temperature [RT]). After centrifugation (30 min, 4°C, maximum speed), the supernatant containing the solubilized proteins was transferred to a fresh tube and the protein concentration was measured using a standard Bradford assay (Bio-Rad) and adjusted to a final concentration of 1 mg/ml. To reduce and alkylate disulfide bonds, 1 µl TCEP [tris(2-carboxyethyl)phosphine, 0.2 M stock in 0.1 M Tris, pH 8.5] was added to 40 µl protein extract (37°C, 1 h, 1,000 rpm). After the samples were cooled down, 1 µl fresh iodoacetamide solution (0.4 M stock in high-pressure liquid chromatography [HPLC] water) was added and incubated in the dark (25°C, 30 min, 500 rpm). Finally, 1 µl *N*-acetyl-cysteine solution (0.5 M stock in 0.1 M Tris, pH 8.5) was added, vortexed, and incubated (RT, 500 rpm, 10 min). For the proteolysis, Lys-C (0.2-µg/µl stock; Wako) was added to a final enzyme/protein ratio of 1:100 (37°C, 4 h, 550 rpm). The sample was diluted 1:5 (vol/vol) to a final urea concentration below 2 M using fresh 0.1 M ABC buffer (ammonium bicarbonate in HPLC water). Porcine trypsin (0.4-µg/µl stock; Promega) was added to a final trypsin/protein ratio of 1:50 (37°C, ON, 550 rpm). Postdigestion, trifluoroacetic acid (TFA; 5% stock in HPLC water) was used to decrease the pH below 2. For the solid-phase extraction, C_18_ microspin columns (Harvard Apparatus) were conditioned with 150 µl acetonitrile (2,400 rpm, 30 s) and equilibrated twice with 150 µl TFA (0.1% stock in HPLC water, 2,400 rpm, 30 s). The sample was transferred twice through the column (2,000 rpm, 2 min) before the column was washed 5 times with 150 µl wash buffer (5% acetonitrile, 95% HPLC water, and 0.1% TFA) (2,400 rpm, 30 s). The peptides were eluted twice with 150 µl elution buffer (50% acetonitrile, 50% HPLC water, and 0.1% TFA) and concentrated under vacuum to dryness using a tabletop concentrator (Eppendorf). The peptides were dissolved to a final concentration of 0.5 µg/µl in liquid chromatography-tandem mass spectrometry (LS-MS/MS) buffer (0.15% formic acid, 2% acetonitrile, HPLC water) using 20 pulses of ultrasonication (Vial Tweeter; Hielscher) (amplitude 100, cycle 0.5) and shaking in a Thermocycler (37°C, 5 min, 1,400 rpm) (Eppendorf).

### Coimmunoprecipitation analysis.

Independent cultures of C. crescentus strains UJ5511 and UJ6643 were grown in PYE to an OD_660_ of 0.3. Cells were pelleted, washed twice in 50 ml of 20 mM Tris (pH 8.0)-100 mM NaCl, and resuspended in 10 ml Bug Buster (Novagen) supplemented with 1 µl Complete mini-protease inhibitor (Roche), 200 µg/ml lysozyme, and Benzonase (0.5 µl/ml). After incubation at room temperature (20 min, gentle shaking), cell debris was removed by centrifugation (10,000 × *g*, 15 min, 4°C). One hundred fifty microliters Protino nickel-nitrilotriacetic acid (Ni-NTA) agarose (Macherey-Nagel) was washed 3 times in 500 µl Bug Buster (1,000 × *g*, 1 min, 4°C) and incubated with the cleared lysate (ON, 4°C, 10 rpm on a rotary wheel). The beads were transferred to a BioSpin column (Bio-Rad) and washed 4 times with 700 µl HNN lysis buffer (50 mM HEPES, pH 7.5, 150 mM NaCl, 50 mM NaF, 5 mM EDTA) with 0.5% IGEPAL CA-630 (Sigma-Aldrich), before being washed 4 times with HNN lysis buffer without detergent. The protein extract was eluted using 3 washes with 150 µl of 0.2 M glycine (in HPLC water, pH 2.5). The eluate was neutralized with 150 µl ABC buffer (ammonium bicarbonate, 1 M stock in HPLC-grade water). Urea (8 M stock in 100 mM ABC buffer) was added to a final concentration of 1.6 M, and the sample was vortexed before reducing and alkylating of disulfide bonds as follows. One microliter of TCEP [tris(2-carboxyethyl)phosphine, 0.2 M in 100 mM ABC buffer] was added per 40 µl protein extract (37°C, 30 min, 1,000 × *g*). After cooling down, 1 µl fresh iodoacetamide (0.4 M stock in HPLC water) was added per 40 µl protein extract and incubated in the dark (25°C, 30 min, 500 rpm). Finally, 1 µl *N*-acetyl-cysteine solution (0.5 M in 0.1 M ABC buffer) was added per 40 µl sample, vortexed, and incubated (25°C, 10 min, 500 rpm). For proteolysis, 1 µg porcine trypsin (0.4-µg/µl stock, Promega) was added (ON, 37°C, 500 rpm). For peptide purification, 150 µl TFA (trifluoroacetic acid; 5% stock in HPLC water) was added to decrease the pH below 3. C_18_ microspin columns (Thermo Scientific) were conditioned twice with 150 µl acetonitrile (1,600 rpm, 30 s) and equilibrated 3 times with 150 µl 0.1% TFA (2,400 rpm, 30 s). The samples were loaded, and the flowthrough was collected in a fresh tube (1,800 rpm, 2 min). The flowthrough was reloaded and centrifuged again (1,800 rpm, 2 min). A mixture of 5% acetonitrile, 95% (vol/vol) HPLC water, and 0.1% TFA was used to wash the columns 3 times with a 150-µl volume (2,400 rpm, 30 s). Bonded peptides were eluted into a new tube 3 times using 100 µl elution buffer (50% acetonitrile, 50% [vol/vol] HPLC water, and 0.1% TFA) (1,600 rpm, 30 s). A SpeedVac (Eppendorf) was used to concentrate the eluted peptide mixture to dryness. The peptides were dissolved in 50 µl LC buffer A (0.15% formic acid, 2% acetonitrile) using 20 pulses of ultrasonication (Vial Tweeter; Hielscher) (amplitude 100, cycle 0.5) and shaking (25°C, 5 min, 1,400 rpm).

### Yeast two-hybrid screening.

S. cerevisiae PJ69-4A (UJ5292) (67) was transformed with pIDJ041 (UJ6743). Single colonies of S. cerevisiae PJ69-4A containing the bait plasmid pIDJ041 were used for library-scale transformation with a C. crescentus library (68). Transformants (2.2 × 10^6^) were screened on plates lacking histidine (SC-Trp-Leu-His plus 5 mM 3′AT), and single colonies were used to isolate prey plasmids for sequencing.

### Protein purification.

E. coli BL21 containing plasmids of interest was grown at 30°C in lysogeny broth (LB) medium and induced with 1 mM isopropyl-β-d-1-thiogalactopyranoside (IPTG) for protein overproduction at an OD_600_ of 0.6. Cells were harvested 2 h after induction (5,000 rpm, 15 min, 4°C) and stored at −80°C. After resuspension in lysis buffer (1× phosphate-buffered saline [PBS], 10 µg/ml DNase, one Complete mini-protease inhibitor tablet [Roche]), cells were disrupted with a French press and the supernatant containing the protein of interest was separated from the cell lysate by centrifugation (11,000 relative centrifugal force [rcf], 1 h, 4°C). The supernatant was incubated with 1 ml Protino Ni-NTA agarose (Macherey-Nagel) (11 rpm, 1 h, 4°C) and was washed with 2× PBS, 500 mM NaCl, 10 mM imidazole (pH 8.0), 1 mM dithiothreitol (DTT). The protein was eluted with 1× PBS, 500 mM NaCl, 250 mM imidazole (pH 8.0), 1 mM DTT and dialyzed in Spectra/POR membranes (Spectrum Laboratories) using 10 mM HEPES-KOH (pH 8.0), 50 mM KCl, 10% glycerol, 0.1 mM EDTA (pH 8.0), 5 mM β-mercaptoethanol, 5 mM MgCl_2_.

### *In vitro* phosphorylation.

Kinase and phosphatase assays were adapted from reference 33. Unless otherwise stated, 5 µM protein concentrations were used. Reaction mixtures were incubated in dialysis buffer in the presence of 500 µM ATP and 2.5 µCi [γ-^32^P]ATP (3,000 Ci mmol^−1^; Hartmann Analytic) at room temperature. Additional proteins were added, and reactions were stopped by the addition of SDS sample buffer at indicated time points. Reaction mixtures were stored on ice or loaded on 12% SDS gels. Wet gels were exposed to phosphor screens (0.5 to 1.5 h) before being scanned using a Typhoon FLA7000 imaging system (GE Healthcare). In experiments assessing phosphatase activity, ATP was depleted by the addition of 1.5 units of hexokinase (Roche) and 5 mM d-glucose 15 min after phosphorylation. For the purification of MrrA~P, the following conditions were used. CC2874 (0.2 µM) and MrrA (100 µM) were prephosphorylated for 1 h. Twenty-five microliters of anti-MBP magnetic beads (New England Biolabs; E8037S) was added and incubated for 1 h. Beads were then concentrated using a magnet. Hexokinase and glucose were added to the supernatant as described above and incubated for 10 min to deplete remaining ATP.

## Note Added after Publication

Fig. S2 and Table S2 in the supplemental material were replaced after publication. The article was corrected online on 30 August 2018. See the Erratum at https://doi.org/10.1128/mBio.01534-18.
