# Erratum for Lori et al., “A Single-Domain Response Regulator Functions as an Integrating Hub To Coordinate General Stress Response and Development in Alphaproteobacteria”

**DOI:** 10.1128/mBio.01534-18

**Published:** 2018-09-04

**Authors:** C. Lori, A. Kaczmarczyk, I. de Jong, U. Jenal

**Affiliations:** a Focal Area of Infection Biology, Biozentrum, University of Basel, Basel, Switzerland

## ERRATUM

Volume 9, issue 3, e00809-18, 2018, https://doi.org/10.1128/mBio.00809-18. We inadvertently referred to Asp53 as the phosphorylated residue in MrrA, instead of Asp58, in the main text (“MrrA is a central phosphorylation hub for multiple histidine kinases”) and in Fig. S2 in the supplemental material. We also listed the wrong primers to construct this mutant version of MrrA. The primers originally listed were indeed for the Asp53Asn mutation, which however was not tested. The construct used and shown in our paper (Fig. S2A) is the Asp58Asn (D58N) mutant version, and we verified this by resequencing the insert part of the plasmid. Accordingly, we have corrected Fig. S2A and Table S2 and they have been replaced online. These changes do not in any way affect the conclusions of the paper. We apologize to the readers for any confusion that may have arisen from our mistake.

