# Supplementary figures and images for: A Single-Domain Response Regulator Functions as an Integrating Hub To Coordinate General Stress Response and Development in Alphaproteobacteria
Source: mBio. 2018 May 22;9(3):e00809-18. doi: 10.1128/mBio.00809-18 (PMC5964349; doi:10.1128/mBio.00809-18)

Figure S1

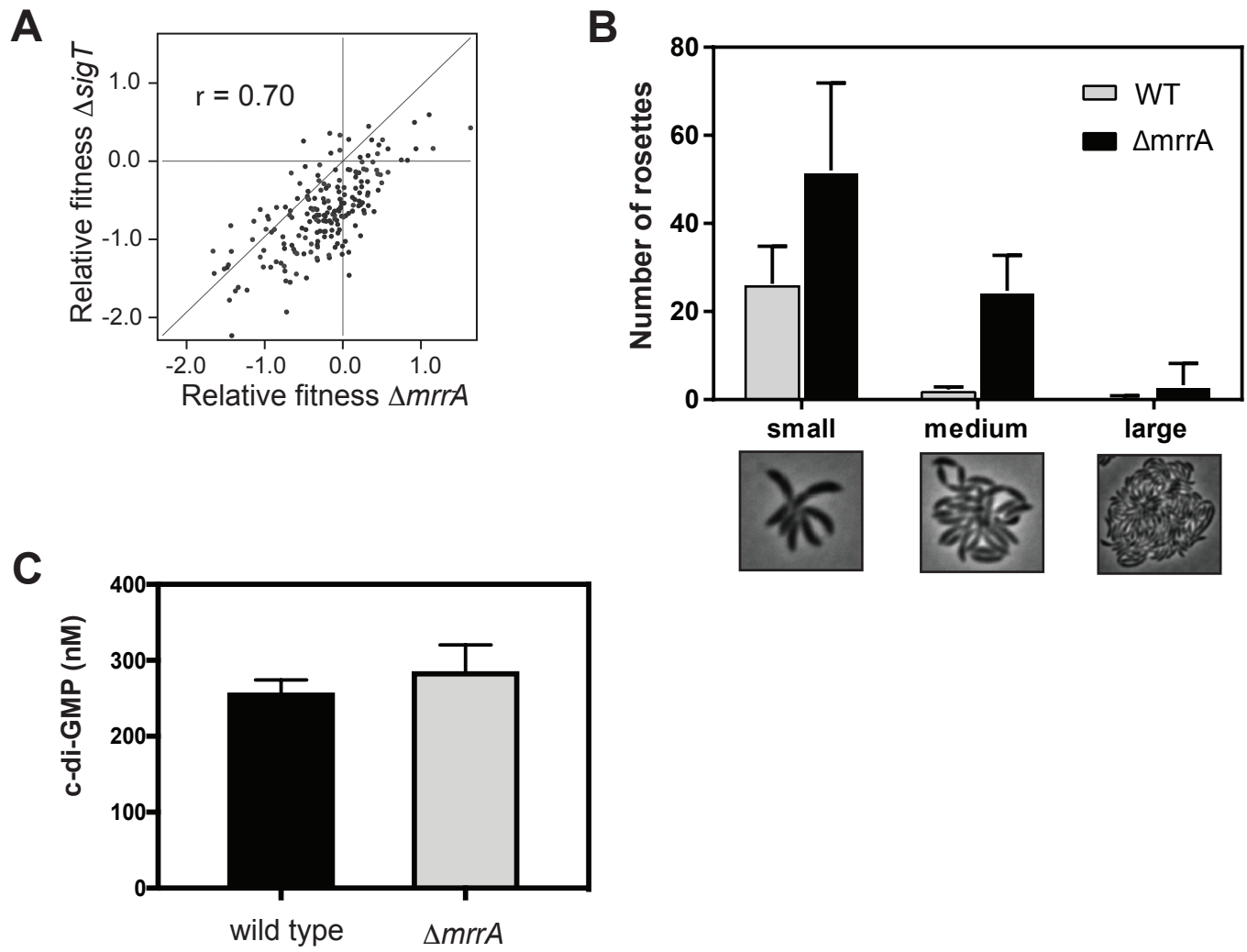

Supplement: FIG S1 [file mbo003183890sf1.pdf]

A

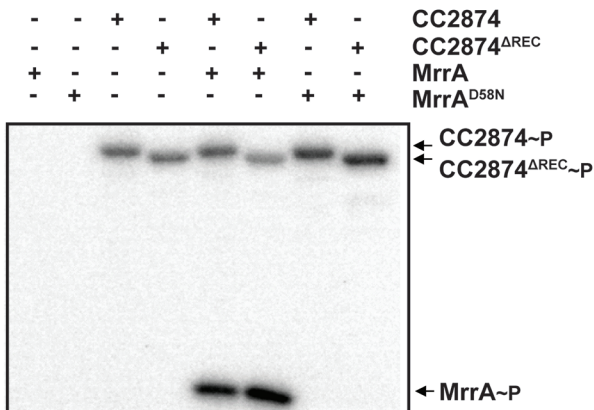

B

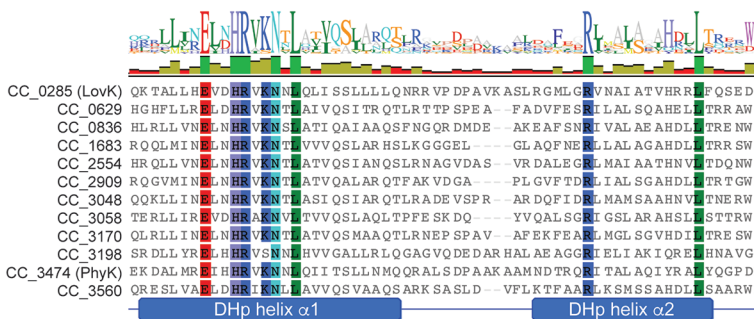

D

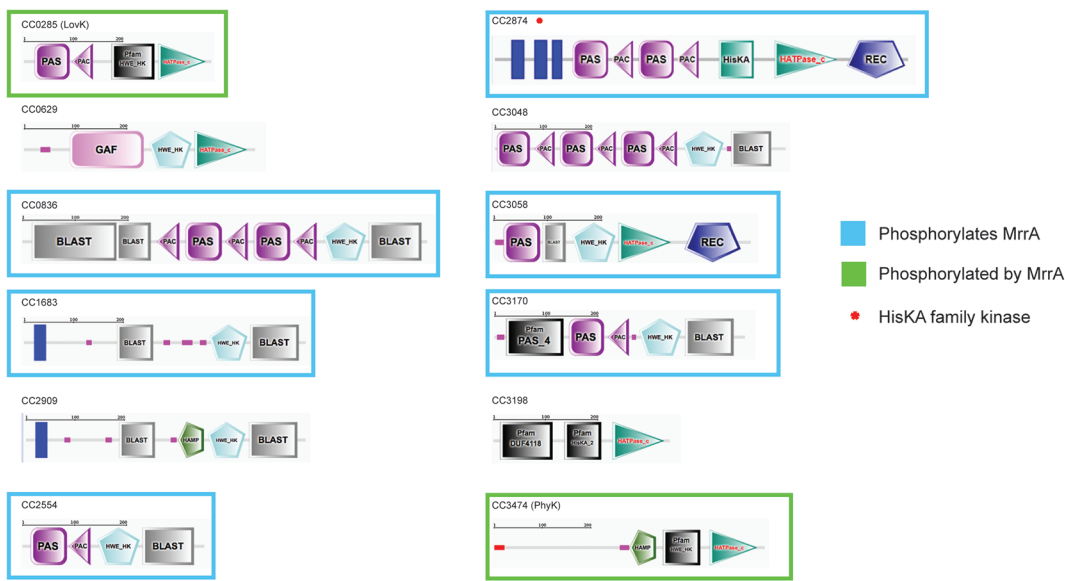

Supplement: FIG S2 [file mbo003183890sf2.pdf]

Figure S3

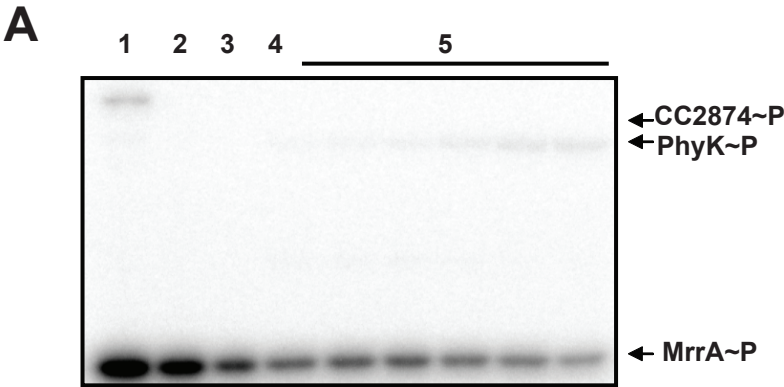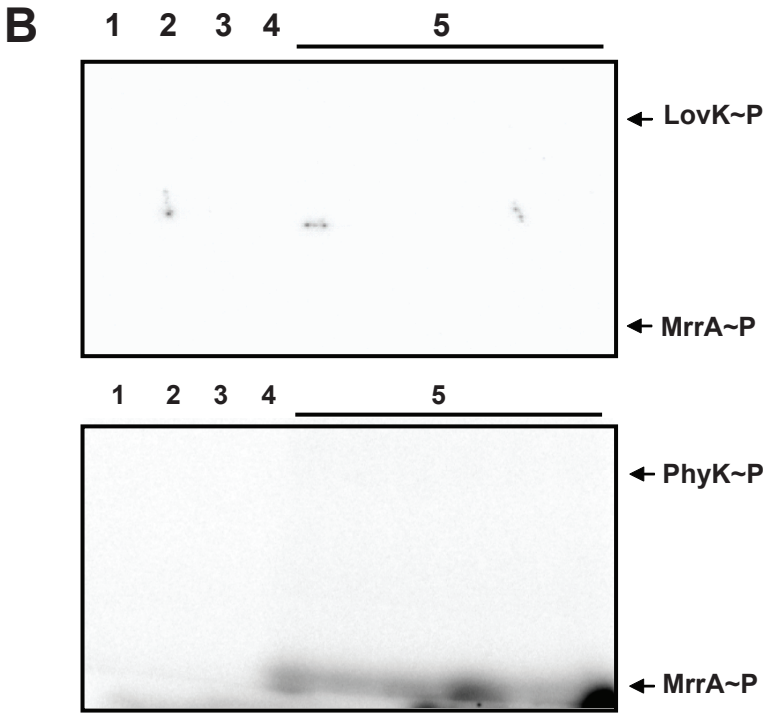

Supplement: FIG S3 [file mbo003183890sf3.pdf]
