# Supplementary material for: A Single-Domain Response Regulator Functions as an Integrating Hub To Coordinate General Stress Response and Development in Alphaproteobacteria
Source: mBio. 2018 May 22;9(3):e00809-18. doi: 10.1128/mBio.00809-18 (PMC5964349; doi:10.1128/mBio.00809-18)
Supplement: TABLE S1 [file mbo003183890st1.pdf]

| A | Protein | Essential | Function | Total spec counts MrrA | Total spec counts MrrA3xFLAG | Fold change (+/- FLAG) | T-test |
|---|---|---|---|---|---|---|---|
| | DgcB | no | signaling | 1 | 11 | 11 | 0.03 |
| | ChpT | yes | signaling | 0 | 9 | 9 | 0.00 |
| | ParE | yes | DNA replication | 0 | 22 | 22 | 0.01 |
| | CC2874 | no | signaling | 0 | 24 | 24 | 0.04 |
| | CC1056 | no | DUF | 1 | 15 | 15 | 0.05 |

| B | Protein | Essential | Function | Transformants |
|---|---|---|---|---|
| | CC2330 | no | transcription factor | 2 |
| | CC2554 | no | kinase | 1 |
| | CC2874 | no | hybrid kinase | 5 |

| C | Protein | Essential | Function | Ratio (Δ*mrrA*/wt) |
|---|---|---|---|---|
| | CC2576 | no | SD-RR | 0.25 |
| | CC3147 | no | TonB-dep receptor | 4.34 |
| | MucRIII | no | transcription factor | 0.02 |
| | NepR | yes | anti-sigma factor | 0.10 |
| | PhyR | no | anti-anti-sigma factor | 0.22 |
| | SigT | no | sigma factor | 0.08 |
